# Next Generation Aqueous Two‐Phase System for Gentle, Effective, and Timely Extracellular Vesicle Isolation and Transcriptomic Analysis

**DOI:** 10.1002/jev2.70058

**Published:** 2025-03-19

**Authors:** Boyang Su, Morteza Jeyhani, Gobi Thillainadesan, Minzhi Sheng, Reese Wunsche, Thamara Dayarathna, Kristin Cimolai, Hanyi Weng, Katarzyna J. Jerzak, Stanley K. Liu, Scott S. H. Tsai, Hon S. Leong

**Affiliations:** ^1^ Biological Sciences Platform Sunnybrook Research Institute Toronto Ontario Canada; ^2^ Temerty Faculty of Medicine, Department of Medical Biophysics University of Toronto Toronto Ontario Canada; ^3^ Department of Mechanical, Industrial, and Mechatronics Engineering Toronto Metropolitan University Toronto Ontario Canada; ^4^ Keenan Research Centre for Biomedical Science St. Michael's Hospital Toronto Ontario Canada; ^5^ Institute for Biomedical Engineering, Science and Technology (iBEST)—Toronto Metropolitan University and St. Michael's Hospital Toronto Ontario Canada; ^6^ Department of Electrical, Computer and Biomedical Engineering Toronto Metropolitan University Toronto Ontario Canada; ^7^ Division of Medical Oncology and Hematology, Department of Medicine Odette Cancer Centre Sunnybrook Health Sciences Centre Toronto Ontario Canada; ^8^ Department of Radiation Oncology University of Toronto Toronto Ontario Canada

**Keywords:** biomarker analysis, circRNA, dextranase, EV isolation, extracellular vesicles (EVs), miRNA, nanoscale flow cytometry, next‐generation aqueous two‐phase system (next‐gen ATPS), RNA extraction, snoRNA

## Abstract

The isolation of extracellular vesicles (EVs) using currently available methods frequently compromises purity and yield to prioritize speed. Here, we present a next‐generation aqueous two‐phase system (next‐gen ATPS) for the isolation of EVs regardless of scale and volume that is superior to conventional methods such as ultracentrifugation (UC) and commercial kits. This is made possible by the two aqueous phases, one rich in polyethylene glycol (PEG) and the other rich in dextran (DEX), whereby fully encapsulated lipid vesicles preferentially migrate to the DEX‐rich phase to achieve a local energy minimum for the EVs. Isolated EVs as found in the DEX‐rich phase are more amenable to biomarker analysis such as nanoscale flow cytometry (nFC) when using various pre‐conjugated antibodies specific for CD9, CD63 and CD81. TRIzol RNA isolation is further enabled by the addition of dextranase, a critical component of this next‐gen ATPS method. RNA yield of next‐gen ATPS‐isolated EVs is superior to UC and other commercial kits. This negates the use of specialized EV RNA extraction kits. The use of dextranase also enables more accurate immunoreactivity of pre‐conjugated antibodies for the detection of EVs by nFC. Transcriptomic analysis of EVs isolated using the next‐gen ATPS revealed a strong overlap in microRNA (miRNA), circular RNA (circRNA) and small nucleolar RNA (snoRNA) profiles with EV donor cells, as well as EVs isolated by UC and the exoRNeasy kit, while detecting a superior number of circRNAs compared to the kit in human samples. Overall, this next‐gen ATPS method stands out as a rapid and highly effective approach to isolate high‐quality EVs in high yield, ensuring optimal extraction and analysis of EV‐encapsulated nucleic acids.

## 1 Introduction

The classic method for extracellular vesicle (EV) isolation is ultracentrifugation (UC), in which EVs in a complex liquid mixture are sedimented by centrifugal force exceeding 100,000 × *g*’s (Gardiner et al. 2016; Royo et al. 2020). UC has been the gold standard method for EV isolation since its first published description in 1960 (Beams et al. 1960). The main drawbacks of UC are excessive damage to the EVs, the extensive time required to pellet EVs and difficulty in identifying or locating the pellet (Théry et al. 2018; Welsh et al. 2024; Linares et al. 2015). New methods in EV isolation, such as commercial kits, partially overcome these drawbacks but are expensive, offer questionable EV yield and do not eliminate soluble proteins effectively (Welsh et al. 2024).

While UC and commercial kits hinder the optimal functionality of EVs, achieving high levels of purity and concentration of EVs is essential for the advancement of EV‐based biomarker development and EV‐based therapeutics (Das et al. 2024; Xu et al. 2016). EV purity requires the removal of major contaminants such as lipoproteins, soluble proteins and viruses (Théry et al. 2018; Welsh et al. 2024), while simultaneously preserving the membrane integrity of EVs is essential for enabling the physiologic function of EVs. Preserving EV cargo such as protein and nucleic acids is essential for EV‐based liquid biopsy and therapeutics development (Maroto et al. 2017; Kumeda et al. 2017).

Here, we developed a next‐generation aqueous two‐phase system (next‐gen ATPS) to isolate EVs. The discovery of first‐generation ATPS was conceived accidentally in 1896 by Martinus Willem Beijerinck (Grilo et al. 2016) and its potential applications in biotechnology were later identified by Per‐Åke Albertsson (Albertsson 1986; Albertsson 1970). These water‐based biphasic systems are a mixture of two incompatible polymers in an aqueous medium, which, at sufficiently high concentrations, separates into two different and distinct phases. One commonly studied ATPS is a combination of polyethylene glycol (PEG) and dextran (DEX) (Hatti‐Kaul 2001). This form of ATPS, which has low interfacial tension and high water content, is biocompatible and has been applied in various areas in biotechnological and biomedical applications, such as separating cells into subpopulations (Walter et al. 1991; Walter and Johansson 1994; Walter et al. 1976; Walter et al. 1980), partitioning biomolecules (Michalski et al. 1986; Nam et al. 2005; SooHoo and Walker 2009; Yamada et al. 2004; Pinilla et al. 1994; Johansson et al. 1985; Cole 1991), creating cell patterns (Tavana et al. 2010; Tavana et al. 2009) and purifying/extracting proteins (Boland 2002; Jordan and Vilter 1991; Grindrod 1970; Mathiazakan et al. 2016; Sikdar et al. 1991). ATPS‐based isolation strategies are cost‐effective, simple to implement, easily scalable and versatile. Theoretically, ATPS is capable of isolating EVs of all types (Kang et al. 2017).

By adjusting the concentrations of the two polymers in an ATPS, it is possible to selectively partition EVs into one of the phases, away from other cellular contaminants and debris. Kim et al. (2015) used ATPS to isolate melanoma‐derived EVs from a mixture of EVs and serum proteins. The authors also verified the diagnostic applicability of the isolated EVs and detected mRNA derived from melanoma cells and CD81 in isolated EVs. Later, Shin et al. (2018) utilized the same method of using ATPS for EV isolation from urine samples of 20 prostate cancer patients and observed a higher quality and quantity of EVs isolated from patients’ samples for diagnostic purposes. However, a major drawback arises when dextran, mixed with alcohol (e.g. isopropanol or ethanol), which is required for phenol‐based RNA extraction (e.g., TRIzol), leads to visible coagulation of the polysaccharide, thus interfering with RNA isolation. This visible coagulation is a major barrier for ATPS being used broadly in the EV field. DEX also potentially introduces artifacts of non‐specific binding between pre‐conjugated antibodies and EVs, thus overestimating EV counts analysed by nanoscale flow cytometry (nFC).

In this paper, innovations in the ATPS method were made leading to the isolation of EVs that are amenable to TRIzol RNA extraction and immunolabeling of EVs for downstream analyses. This was made possible by using dextranase to enzymatically remove DEX in the EV preps. This improvement results in a next‐generation version of ATPS (next‐gen ATPS) that combines the previous benefits of rapid, efficient, and gentle EV isolation with the new advantages of RNA extraction and immunolabeling of EVs.

## 2 Materials and Methods

### 2.1 Sample Collection and Reagents

The study was approved by the Research Ethics Board of Sunnybrook Health Sciences Centre, and written informed consent from all participants was obtained. These were patients (*N* = 13) diagnosed with clinically significant prostate cancer (i.e., Gleason Group ≥ 2). Whole blood samples were collected in K2‐EDTA Vacutainers (BD Biosciences) and centrifuged at 2500 × *g*’s for 15 mins. Plasma samples were removed and stored at −80°C.

### 2.2 Cell Culture

BPH cells were generously obtained from Dr. Simon W. Hayward (Vanderbilt University) and maintained in Roswell Park Memorial Institute (RPMI)‐1640 medium (Wisent Bio Products) supplemented with 5% fetal bovine serum (FBS, Wisent Bio Products). PC3 cells were obtained from ATCC (CRL‐1435) and maintained in Dulbecco's Modified Eagle Medium (DMEM) (Wisent Bio Products) supplemented with 5% FBS. HEK293T cells were obtained from ATCC (CRL‐3126) and maintained in DMEM supplemented with 5% FBS. MDA‐MB‐231 cells were obtained from ATCC (CRL‐3126) and maintained in DMEM supplemented with 10% FBS. All cells were grown in a 37°C tissue culture incubator at 5% CO_2_.

### 2.3 Lentivirus Generation

The lentivirus plasmid pLVX‐ZsGreen1‐N1 (Takara Bio, 632565) was co‐transfected into HEK293T cells with two other plasmids, pMD2.G (Addgene, 12259) and psPAX2 (Addgene, 12260), which express vesicular stomatitis virus (VSV) envelope protein and human immunodeficiency virus (HIV) packaging protein, respectively. Self‐inactivating lentivirus was collected from the conditioned media 72 hr post‐transfection. The conditioned media was centrifuged at 200 × *g*’s, filtered through a 0.22‐µm filter (FroggaBio Inc.) and cryopreserved at −80°C.

### 2.4 Lentivirus Transduction

2 × 10^5^ host cells were seeded on six‐well plates 24 hr before transduction. Five hundred microlitres of lentivirus stock was co‐cultured with cells in the presence of 9‐µg/mL Polybrene (Santa Cruz Biotechnology) for 48 hr. Transduced cells were further treated with 1.5‐µg/mL puromycin (Wisent Bio Products) to select transgenic cells with stable zsGreen fluorescent protein expression. Transduced cells were grown in selective media with 0.25‐µg/mL puromycin for long‐term maintenance.

### 2.5 *In Vitro* Generation of Extracellular Vesicles (EVs)

EV donor cells were grown to 80%–100% confluence and washed with phosphate‐buffered saline (PBS, Wisent Bio Products), followed by starvation in serum‐free growth media for 72 hr. Cell culture conditioned media was then collected and centrifuged at 2000 × *g*’s for 10 mins to remove dead cells and cell debris. Supernatant (conditioned media) was cryopreserved at −80°C for subsequent EV isolation and analyses.

### 2.6 Centrifugation and Ultracentrifugation of EVs

Cell culture conditioned media was thawed at 37°C and centrifuged at 2000 × *g*’s for 10 mins to remove large cell debris before UC. UC was conducted with a Beckman Optima XPN‐80 UC machine fitted with a SW 41 Ti rotor at 200,000 × *g*’s for 2 h at 4°C. Supernatant was removed by aspiration and inverting UC tubes (Beckman Coulter Inc., 331372) on Kimwipes. EV pellets were dissolved in PBS and stored at −80°C for subsequent analyses.

### 2.7 Aqueous Two‐Phase System and Dextranase Treatment

PEG (Mw 35 kDa, Sigma‐Aldrich) and DEX (Mw 500 kDa, Pharmacosmos, Denmark) stocks were dissolved in PBS. Cell culture conditioned media or human plasma samples were mixed with PEG and DEX stocks to final concentrations of 3% (weight by volume, *w*/*v*) and 1.5% (*w*/*v*), respectively, in 1.2‐, 6‐ or 12‐mL configurations. PBS was used to adjust the total volume as needed. Phase separation was performed by centrifuging at 200 × *g*’s for 15 mins, with the PEG‐rich phase at the top (approximately 90% by volume) and the DEX‐rich phase at the bottom (approximately 10% by volume). PEG‐ and DEX‐rich phases were separated by micropipetting. The EV‐containing DEX‐rich phase was cryopreserved at −80°C for long‐term storage. Dextranase treatment was performed by mixing dextranase from *Chaetomium erraticum* (Sigma‐Aldrich) with the EV‐containing DEX‐rich phase at designated concentrations (volume by volume, *v*/*v*). Mixtures were then vortexed and incubated at 37°C for 30 mins to facilitate the enzymatic reaction. This dextranase‐treated DEX‐rich phase is then amenable to downstream EV analyses such as nFC and RNA extraction.

### 2.8 Nanoscale Flow Cytometry of EVs

EVs were analysed by the A60Micro‐Plus nanoscale flow cytometer (Apogee Flow Systems Inc., United Kingdom). Fluorescence was detected by 405‐, 488‐ and 639‐nm illumination wavelengths, respectively. Photomultiplier tube (PMT) voltages were set as follows: LALS (325 V), SALS (355 V), 488‐Grn (425 V, for zsGreen) and 488‐Org (425 V, for Phycoerythrin, PE). On the control panel, the sheath pressure was set to 200 mbar, the sample flow rate was set to 1.50 µL/min and the acquisition time was set to 30 s. To label EVs with antibodies against membrane biomarkers, 50 ng of each antibody was mixed with 10 µL of EV‐containing fluids, incubated in dark for 30 mins, diluted with PBS and then analysed by nFC. Antibodies used in this study include PE mouse anti‐human CD9 (BD Biosciences, 555372), PE mouse anti‐human CD63 (BD Biosciences, 561925), PE mouse anti‐human CD81 (BD Biosciences, 561957) and PE mouse IgG1 k isotype control (BD Biosciences, 555749). zsGreen‐packaged EVs were directly analysed with the same settings. Graphs were rendered comparing long‐angle light scatter (LALS) to the fluorescent intensity and short‐angle light scatter (SALS) separately. The ApogeeMix size calibration beads (Apogee Flow Systems Inc., 1527) were used to determine sizes of EVs.

### 2.9 Transmission Electron Microscopy (TEM)

TEM analysis was performed at the Cellular and Molecular Electron Microscopy core of Peter Gilgan Centre for Research and Learning using a FEI Techai 20 electron microscope at direct magnification of 80,000–150,000 times. EV samples were adsorbed onto charged carbon grids (Electron Microscopy Sciences) for 5 mins and stained with 2% uranyl acetate for 40 s. Stained EVs were washed with MilliQ water prior to imaging.

### 2.10 EV Uptake Assay and Confocal Microscopy

Recipient cells were seeded on cover slides in 12‐well plates and treated with 100 million zsGreen expressing EVs (purified by ATPS or UC) that were pre‐analysed by nFC. After 24‐ and 48‐hr incubation, recipient cells were washed with PBS, fixed with formalin, stained with Hoechst 33342 (nucleus stain, Thermo Fisher Scientific, 2134015) and Wheat Germ Agglutinin (WGA) Alexa Fluor 647 (membrane stain, Thermo Fisher Scientific, 2199103) and mounted (Agilent, S3023) for confocal microscopy. Z‐stacks were taken using confocal microscopy (Nikon A1R MP). *XY* and *ZY* views were analysed using NIS Element Analysis software. The proportion of recipient cells with zsGreen signals was counted (*n* = 100).

### 2.11 RNA Isolation

EV RNA isolation in this study was conducted using the TRIzol‐choloroform method followed by isopropanol precipitation or purification using the Qiagen exoRNeasy Midi Kit, following the manufacturer's protocol. For ATPS‐isolated EVs after dextranase treatment, the ratio between the volume of EV samples, TRIzol (Thermo Fisher Scientific) and chloroform (Fisher Scientific) was 1:5:1. The top aqueous layer after TRIzol‐chloroform separation was mixed with isopropanol (Sigma‐Aldrich), 1:1 in volume. RNA was precipitated overnight at −20°C. For UC‐isolated EVs, EV pellets were directly mixed with TRIzol. RNA isolation from human cells was conducted using the same TRIzol method. RNA quality was accessed using a 2100 Bioanalyzer (Agilent Technologies Inc.) at the Genomics Core Facility of Sunnybrook Research Institute.

### 2.12 Cell‐Free RNA Depletion Assay

Ten micrograms of total RNA isolated from PC3‐zsGreen cells was mixed with 1‐mL PC3‐zsGreen CM or RNase‐free water. For the RNase treatment, PureLink RNase A (Thermo Fisher Scientific) was used with a final concentration of 0.1 ng/µL and incubated at 37°C for 30 min. All samples in the cell‐free RNA depletion experiment were processed with ATPS, followed by dextranase treatment, TRIzol RNA isolation and analysed with 2100 Bioanalyzer.

### 2.13 RT‐qPCR

Strand‐specific reverse transcription was conducted using the ProtoScript II Reverse Transcription system (New England BioLabs) with gene‐specific reverse primers (Table S1). cDNA was diluted five times before performing qPCR with Advanced qPCR Master Mix (Wisent Bio Products) on a CFx Real‐Time System (Bio‐Rad Laboratories). PCR condition was 40 cycles of 95°C for 10 s and 65°C for 30 s followed by a melting curve stage. Relative copy number of target RNA was quantified using the ∆Ct method by setting the reference Ct to 40 cycles. Each reaction was run in triplicate and the data show results taken from three independent runs.

### 2.14 Small RNAseq

EV RNA isolated from conditioned media of PC3 and MDA‐MB‐231 cell lines and prostate cancer patient plasma samples using next‐gen ATPS/UC followed by TRIzol and exoRNeasy kit were submitted for small RNA sequencing at PrimBio Research Institute. Cell total RNA from EV donor cells were also submitted. Small RNA sequencing libraries were prepared using NEBNext Multiplex Small RNA Library Prep Set for Illumina kit (NEB#E7560S), following the manufacturer's instruction. Briefly, RNA from each sample was ligated to 3′SR adaptor, followed by hybridizing to reverse transcription primers to prevent adaptor‐dimer formation. Then the 5′SR was ligated to the RNA, and the reverse transcription was conducted using ProtoScript II Reverse Transcriptase, with the presence of Murine RNase Inhibitor. The cDNA generated was amplified using LongAmp Taq 2x Master Mix and sample‐specific index primers. The final library with a size around 140–150 bp was purified with AMpureXP beads (Beckmen Coulter, A63882) and quantified with Agilent 2100 Bioanalyzer High Sensitivity DNA Analysis. All final libraries were normalized to a concentration of 750 pM and pooled. About 20 µL of pooled library was loaded to a P2 flow cell/100 cycle cartridge, and then run on an Illumina NextSeq 2000 sequencer. The raw sequencing data were de‐multiplexed and processed with Illumina DRAGEN BCL Convert 3.8.4.

### 2.15 Small RNAseq Bioinformatics

Quality control of sequencing reads was performed by discarding those with a quality score below Q20 on a 4 bp sliding window. Size selection was subsequently applied, retaining reads ranging from 18 to 35 base pairs in length. Multimapping reads were considered with more weight given to unique mapping reads. Alignment was done using STAR aligner to the hg38 human genome, and annotation of the small RNAs was conducted using the Comprehensive Platform for Small RNA‐Seq data Analysis (COMPSRA). Normalization of the reads was executed by converting the raw counts to Transcripts Per Million (TPM) to facilitate comparative analysis across different sample groups. Small RNAs were annotated into categories such as microRNA (miRNA), circular RNA (circRNA), and small nucleolar RNA (snoRNA).

### 2.16 Immunoblotting

EV protein and protein contamination levels in EV preparation after the application of ATPS on human samples were assessed using immunoblotting analysis. EVs were lysed by mixing 2x RIPA buffer with EV samples (1:1 by volume), in the presence of 1% of protease and phosphatase inhibitor (Thermo Fisher Scientific), followed by incubation on ice for 30 mins. Protein lysates were mixed with 4x Laemmli sample buffer (Bio‐Rad Laboratories), boiled at 95°C for 10 mins and separated by SDS‐PAGE on 4%–20% Tris‐Glycine gels (Invitrogen). Proteins were transferred onto 0.2‐µm Hydrophobic PVDF membranes (MilliporeSigma) post‐gel‐electrophoresis, followed by blocking in 5% skim milk for 1 hr at room temperature. Primary antibodies against EV marker CD9 (BD Biosciences, cat#555370, 1:200) and lipoprotein contamination marker Apolipoprotein A1 (ApoA1, Abcam, ab52945, 1:1000) were incubated with membranes overnight at 4°C. HRP‐conjugated secondary antibodies (Abcam, ab205718 and ab6789, 1:6000) targeting appropriate host species of primary antibodies were incubated with membranes for 1 hr at room temperature. Exposure was performed in the presence of UltraScence Western Substrate (FroggaBio Inc.) and imaged with ChemiDoc Imaging System (Bio‐Rad Laboratories). Quantitative analysis was performed using ImageJ software (NIH).

### 2.17 Statistical Analyses

Statistical analyses were performed using GraphPad Prism 10 software. Results were expressed as the mean ± SEM. Significance was defined as *p* < 0.05 (*), *p* < 0.01 (**), *p* < 0.001 (***), *p* < 0.0001 (****) and *p* > 0.05 (ns). Two‐tailed unpaired *t*‐tests were performed when comparing two groups. One‐way analysis of variance (ANOVA) followed by Tukey's multiple comparisons tests was performed when comparing multiple groups. Two‐way analysis of variance (ANOVA) followed by Tukey's multiple comparisons tests was performed when comparing the partition of EVs in the DEX‐rich phase and pellet in different scales of ATPS.

## 3 Results

### 3.1 ATPS Offers Effective Recovery and Enrichment of Extracellular Vesicles

**FIGURE 1:**
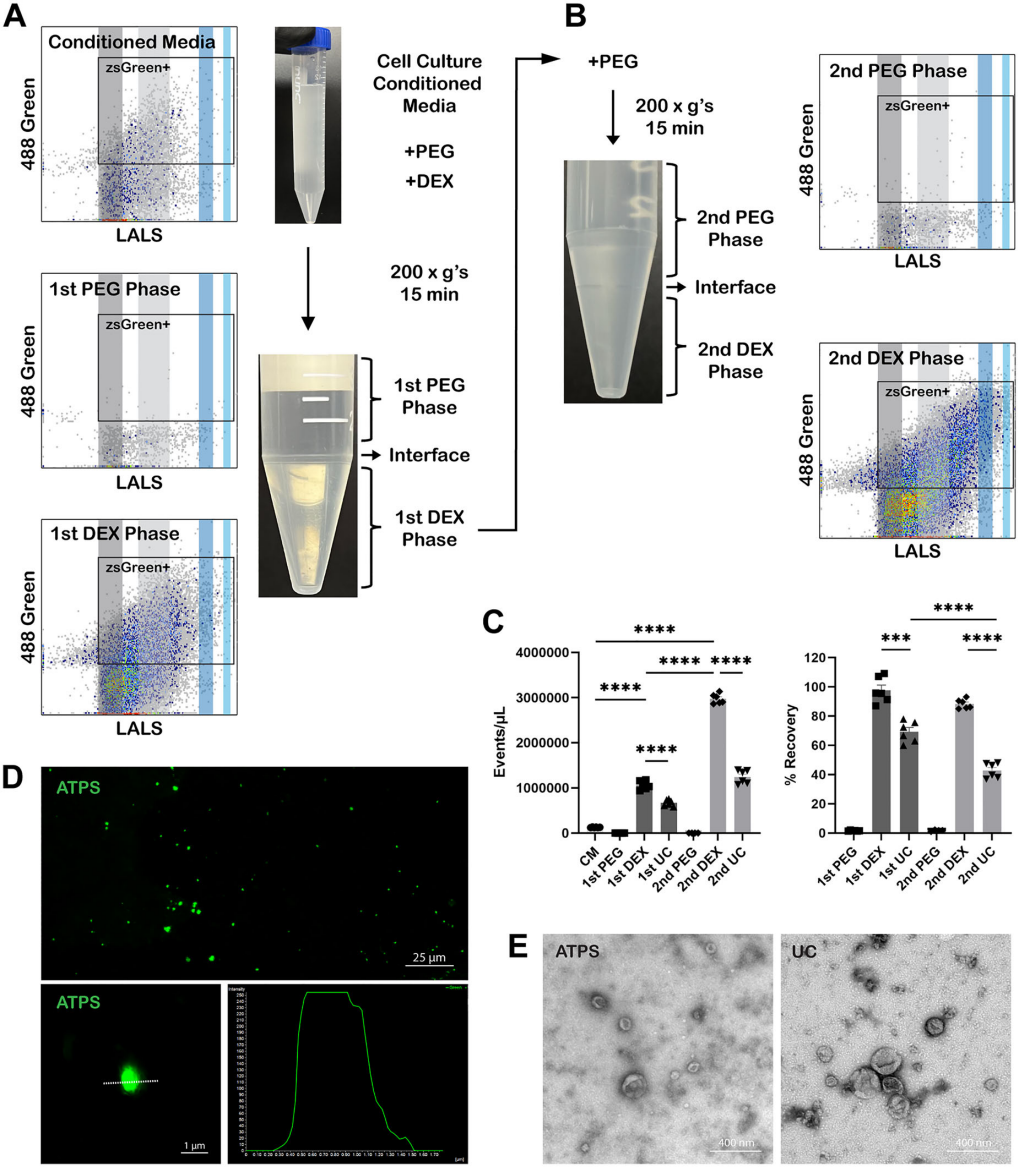
Workflow of a two‐step ATPS and analysis of extracellular vesicles (EV) partition. (A) Schematic diagram of the first step ATPS to isolate EVs from cell culture conditioned media (CM). CM of BPH‐1‐zsGreen cells is mixed with polyethylene glycol and dextran. Mixture is centrifuged at 200 × *g*’s for 15 mins. nFC of CM, 1st PEG‐rich phase and 1st DEX‐rich phase revealed partition and enrichment of EVs in the 1st DEX‐rich phase (left three cytograms). (B) Schematic diagram of the second step ATPS. 1st PEG is removed, and additional polyethylene glycol is mixed with the 1st DEX‐rich phase, followed by centrifugation at 200 × *g*’s for 15 mins. nFC of 2nd PEG‐rich and 2nd DEX‐rich phase revealed partition and greater enrichment of EVs in the 2nd DEX‐rich phase (right two cytograms). (C) EV concentration and recovery efficiency in all phases of two‐step ATPS compared to UC (*n* = 6). (D) Confocal microscopy of the 1st DEX‐rich phase. Scale bars 25 µm (top panel) and 1 µm (bottom left panel). Scan line analysis revealed the size of captured EVs (bottom right panel). (E) TEM analysis of EVs isolated by ATPS (left panel, scale bar 400 nm) and UC (400 nm).

ATPS is formed when two different immiscible polymers that are soluble in an aqueous environment are used at specific concentrations. For example, when PEG (35 kDa) is mixed with DEX (500 kDa) at a ratio of 3%–1.5% (w/v) in an aqueous solution, such as EV‐containing fluid, phase separation occurs and a sharp interface forms between the resulting upper phase (PEG‐rich) and the lower phase (DEX‐rich). This ATPS was applied to cell culture conditioned media (CM) containing EVs from BPH‐1‐zsGreen cells, a cell line transduced with lentivirus for expression of zsGreen fluorescent proteins. The resulting phase‐separated PEG‐rich phase consistently occupies the larger volume upper portion (∼90%), remaining visibly clear, whereas the DEX‐rich phase settles in the smaller volume lower phase, which is 10% of the original mixture by volume (Figure 1A, lower photograph). This separation, with the PEG‐rich phase at the top and the DEX‐rich phase at the bottom, is dictated by the differing densities of the polymers. Using nFC (Figure S1), the concentration of EVs in the starting material can be compared to the concentration of EVs in both the PEG‐ and DEX‐rich phases. After the first round of partitioning with ATPS, no EVs were detected in the PEG‐rich phase and all the EVs were partitioned in the DEX‐rich phase (Figure 1A). EVs in the DEX‐rich phase from the first round of ATPS partitioning can be further submitted to another round of ATPS partitioning by mixing with additional PEG (Figure 1B). As a result, while no EVs were detected in the second PEG‐rich phase, EV concentration in the second DEX‐rich phase was higher than it was in the first DEX‐rich phase, due to the smaller volume of the second DEX‐rich phase.

Figure 1C (left panel) depicts the improvements in EV concentration through two rounds of ATPS partitioning. EV concentration was enriched by ∼8 times and ∼21 times in the first and the second DEX‐rich phase, respectively, in comparison to the EV concentration in the starting material. As a comparison, the starting material was also submitted to two rounds of UC. EV pellets after UC were diluted in PBS with the same volume as the first and the second DEX‐rich phases, respectively. EV concentration was enriched by ∼6 times in the first round of UC and by ∼10 times in the second round of UC, indicating that ATPS has superior EV enrichment capability compared to UC. Figure 1C (right panel) depicts a comparison of the EV recovery efficiency between ATPS and UC. All EVs from the starting material were isolated by the first DEX‐rich phase (∼98% recovery of total EV) and the recovery efficiency was maintained with minimal EV loss in the second DEX‐rich phase (∼88% recovery of total EV). Both UC steps showed lower recovery efficiency than ATPS. Although 69% of total EVs were recovered after the first round of UC, more than half of total EVs were lost after the second round UC (∼43% recovery of total EV). A comparison of ATPS to the Total Exosome Isolation Reagent (TEIR) kit for conditioned media by Invitrogen revealed higher EV concentrations and higher EV recovery rate with the ATPS method (Figures S2A,B). This was achieved within a fraction of the time, which was 20 min with ATPS compared to >12 h when using the TEIR kit for conditioned media samples (Table S2).

Confocal microscopy of ATPS‐isolated EVs revealed zsGreen‐positive EVs that were not aggregated and ranged within 200–1000 nm in diameter (Figure 1D). Scan line analysis of an individual EV (bottom right) confirmed both the size (<800 nm) and vesicular morphology expected of an EV. Transmission electron microscopy (TEM) was also performed to observe the morphology of EVs isolated by ATPS and UC. While ATPS revealed non‐aggregated EVs with intact membrane structures, UC led to the partial aggregation of EVs potentially due to the extreme centrifugal speed and force being applied on EVs during sedimentation (Figure 1E).

### 3.2 Removal of Dextran after ATPS Isolation of EVs Leads to Efficient RNA Extraction

**FIGURE 2:**
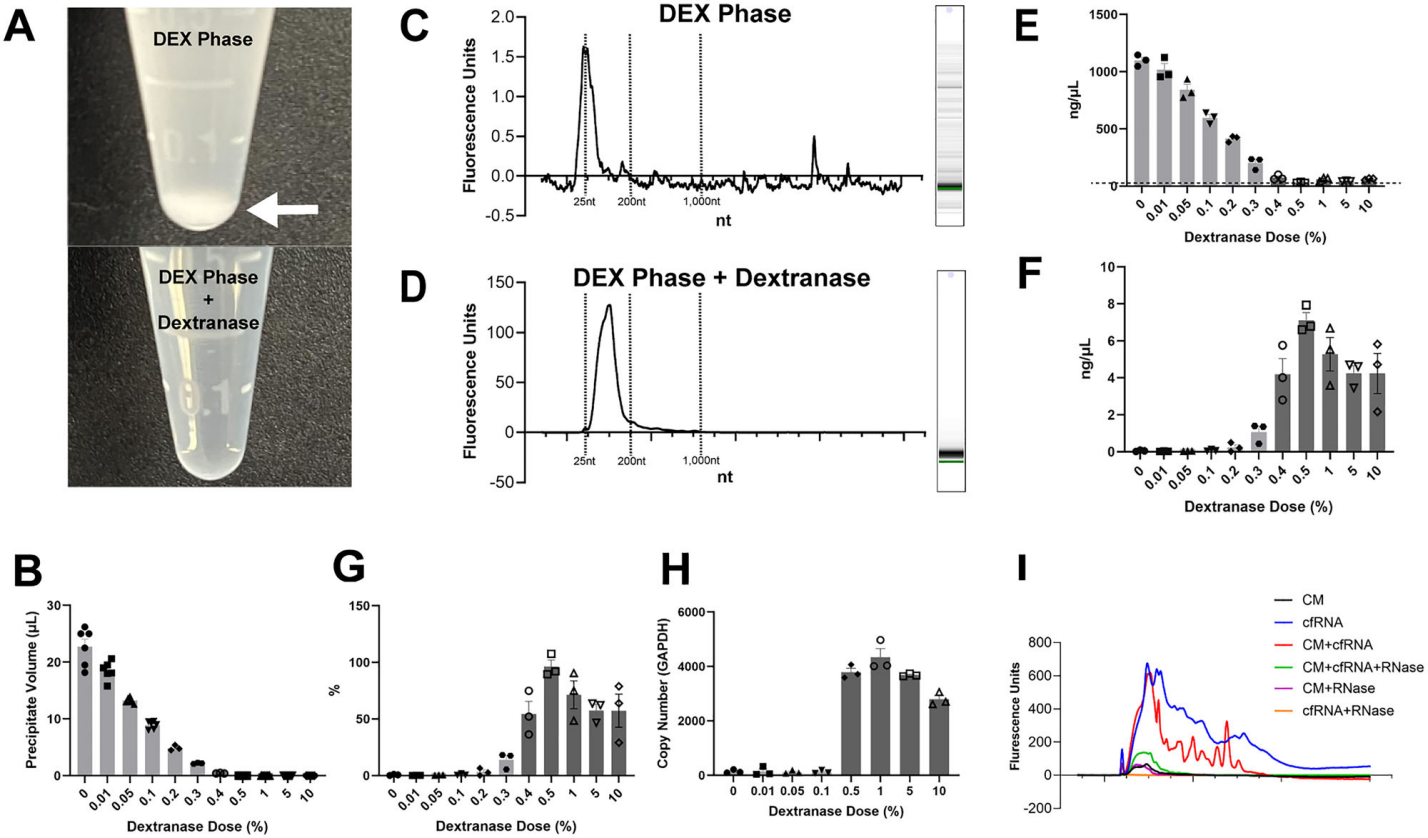
Removal of dextran with dextranase prevents polysaccharide coagulation and leads to efficient RNA extraction. (A) Dextran‐alcohol precipitate is formed during TRIzol RNA isolation with EVs isolated with ATPS (top panel, white arrow). Absence of dextran‐alcohol precipitate during TRIzol RNA isolation after dextranase treatment (bottom panel). (B) Volume of dextran‐alcohol precipitate in EV‐containing DEX‐rich phase treated with dextranase at various concentrations during TRIzol RNA isolation (*n* = 6). (C) Bioanalyzer analysis of EV RNA with dextran‐alcohol precipitate. (D) Bioanalyzer analysis of EV RNA without dextran‐alcohol precipitate after dextranase treatment. (E) NanoDrop measurements of EV RNA isolated from DEX‐rich phase pre‐treated with dextranase at various concentrations (*n* = 3). Dash line represents the expected RNA yield/concentration retrieved from bioanalyzer analysis. (F) Bioanalyzer analysis of concentrations of EV RNA isolated from DEX‐rich phase pre‐treated with dextranase at various concentrations (*n* = 3). (G) Recovery efficiency of RNA isolated from DEX‐rich phase pre‐treated with dextranase at various concentrations (*n* = 3). (H) RT‐qPCR analysis on GAPDH mRNA copy number in RNA isolated from DEX‐rich phase pre‐treated with dextranase at various concentrations (*n* = 3). (I) Bioanalyzer analysis of RNA isolated from CM (black); cfRNA mimicry (blue); RNA isolated from CM with additional cfRNA mimicry (red); RNA isolated from RNase‐treated CM with additional cfRNA mimicry (green); RNA isolated from RNase‐treated CM (purple); RNase‐treated cfRNA mimicry (orange).

An ideal EV isolation method should produce high‐quality EVs amenable for downstream analyses and applications, such as molecular cargo ‘omics’ analyses, flow cytometry, recipient cell/tissue/host treatment and so forth. ATPS isolates EVs with high recovery as shown in Figure 1, but the presence of DEX in the EV‐enriched phase after phase separation leads to minor interference of downstream analyses and applications. In Figure 2, the EV‐containing DEX‐rich phase is not compatible with TRIzol RNA isolation. This high molecular weight polysaccharide co‐precipitates with alcohol (ethanol, isopropanol, etc.) during the RNA precipitation step in TRIzol RNA isolation (Figure 2A, top panel). This highly viscous co‐precipitate not only reduces EV RNA yield but also interferes with downstream analyses such as RNA quantitation. To solve this problem, dextranase, an enzyme that drives hydrolysis reaction of the α‐(1,6)‐glycosidic linkages in DEX (Khalikova et al. 2005), was used to digest the DEX to simpler molecules, such as isomaltose and other oligosaccharides. Complete removal of DEX with a molar excess of dextranase leads to no co‐precipitate contamination during TRIzol RNA isolation (Figure 2A, bottom panel).

To determine the optimal dextranase treatment, the DEX‐rich phase was treated with dextranase at a concentration gradient (0%, 0.01%, 0.05%, 0.1%, 0.5%, 1%, 5% and 10%, *v*/*v*) after ATPS isolation with PC3‐zsGreen CM, followed by TRIzol RNA isolation. DEX‐alcohol co‐precipitate was absent at a minimal dextranase concentration of 0.5% (Figure 2B). The volume of co‐precipitate was negatively correlated with dextranase concentration, indicating the depletion of co‐precipitate was due to DEX degradation by dextranase. Intermediate concentrations between 0.1% and 0.5% for dextranase treatment were also tested (0.2%, 0.3% and 0.4%, *v*/*v*). While the volume of co‐precipitates showed a consistent negative correlation with dextranase concentration, 0.4% dextranase‐treated EVs samples revealed minor but detectable level of co‐precipitation after TRIzol RNA isolation (Figure 2B). Thus, the optimal dextranase concentration for complete depletion of DEX‐alcohol co‐precipitation falls between 0.4% and 0.5%, *v*/*v*.

To analyse and quantify RNA yield, bioanalyzer analysis revealed minimal RNA yield in the samples with dextran‐alcohol co‐precipitate (Figure 2C), while the RNA yield was greatly improved after DEX depletion (Figures 2D and 2F). NanoDrop spectrophotometer is commonly used to measure nucleic acid concentration but was not amenable for RNA samples with DEX‐alcohol co‐precipitates (Figure 2E). The spectrophotometer reading for RNA concentration in samples with co‐precipitates exceeded the expected RNA yield from the number of EVs used for RNA isolation, with the dashed line indicating the expected RNA level as determined by the bioanalyzer analysis (Figure 2E). The spectrophotometer readings were also correlated with the co‐precipitate volume, indicating that this abnormal reading was caused by interference from the co‐precipitates.

RNA recovery was calculated based on TRIzol RNA isolation performed on PC3‐zsGreen CM without ATPS (Figure 2G). DEX‐alcohol co‐precipitates significantly reduced RNA recovery (less than 1%) in samples with insufficient dextranase treatment. Dextranase treatment that depleted DEX‐alcohol co‐precipitates effectively improved RNA recovery to 60%–100%.

RT‐qPCR on GAPDH mRNA was performed on RNA isolated from the DEX‐rich phase treated with multiple dextranase concentrations to validate if the RNA quality is amenable for downstream molecular analysis. Copy number of GAPDH mRNA was found enriched in RNA samples with the absence of DEX‐alcohol co‐precipitate, which approximates RNA concentrations determined in the bioanalyzer analysis (Figure 2H).

It is possible that a portion of RNA isolated from the DEX‐rich phase could be cell‐free RNA (cfRNA) that is not vesicle‐encapsulated. To demonstrate this, we introduced total RNA isolated from PC3‐zsGreen cells to CM as a mimicry of non‐vesicular‐encapsulated cfRNA, prior to ATPS and TRIzol RNA isolation. Bioanalyzer analysis was performed to assess the size distribution of RNA (Figure 2I). When CM was mixed with cfRNA, peaks representing RNA between 200 and 1000 nt were observed (Figure 2I, red), which is larger than the size of EV RNA from CM (25–200 nt, Figure 2I, black). RNase A was used in a depletion assay to degrade any cfRNA not protected by an EV. Upon RNase treatment, large RNA fragments (200–1000 nt) were depleted and only the narrow peak between 25 and 200 bp was observed (Figure 2I, green), representing RNA encased within EVs that resisted RNA degradation. RNase treatment on CM alone did not impact the 25–200 nt EV RNA (Figure 2I, purple), while the treatment on cfRNA alone showed complete depletion (Figure 2I, yellow). These results suggest that ATPS not only isolates RNA from membrane‐encapsulated EVs but also co‐isolates cell‐free RNA.

### 3.3 Dextranase Treatment of Purified EVs via ATPS Prevents Non‐Specific Antibody Binding Resulting in Gentle and Efficient Recovery of EVs

nFC analysis was used to detect EV subpopulations based on membrane biomarkers (Gomes et al. 2018; Padda et al. 2019; Kim et al. 2022). We studied the impact of DEX on immunolabeling with pre‐conjugated antibodies on EVs isolated by ATPS. Various concentrations of DEX were directly mixed with PC3‐zsGreen CM. Pre‐conjugated antibodies targeting general EV biomarkers (CD9, CD63 and CD81) were used to perform the immunolabelling. nFC revealed zsGreen‐positive, biomarker‐positive and zsGreen/biomarker double‐positive EV subpopulations in CM with the presence and absence of DEX (Figure S3A). These subpopulations were further classified as large EVs (lEVs) and small EVs (sEVs) with a size threshold of 100 nm. The concentration of EV subpopulations detected by nFC was dependent on dextran, such as lEVs (Figure S2B), sEVs (Figure S3C) and zsGreen/biomarker double positive subpopulations (Figures S3D and S3E). This demonstrates that DEX interferes with immunolabelling and nFC.

**FIGURE 3:**
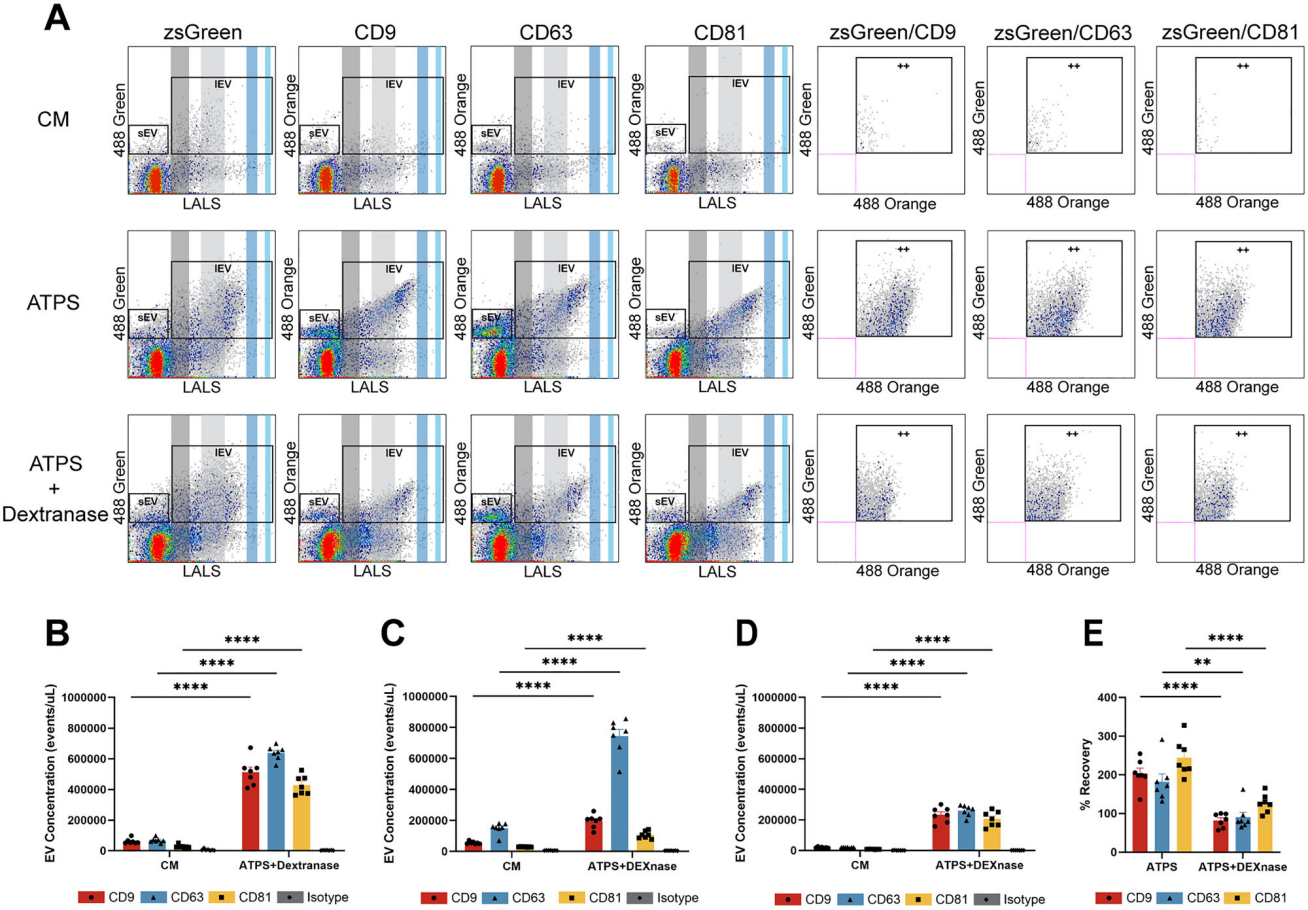
Removal of dextran with dextranase leads to accurate detection of purified EVs by nFC. (A) nFC scatterplots of PC3‐zsGreen CM (top row), ATPS‐isolated PC3‐zsGreen EVs (middle row) and ATPS‐isolated PC3‐zsGreen EVs after dextranase treatment (bottom row). Analysis revealed zsGreen‐positive EV subpopulations (first lane), biomarker‐positive EV subpopulations (second lane to fourth lane) and zsGreen/biomarker double‐positive EV subpopulations (fifth lane to seventh lane). (B) Large EV (lEVs, larger than 100 nm) subpopulation concentration in CM and ATPS‐isolated EVs after dextranase treatment (*n* = 7). (C) Small EV (sEVs, smaller than 100 nm) subpopulation concentration in CM‐ and ATPS‐isolated EVs after dextranase treatment (*n* = 7). (D) zsGreen/biomarker subpopulation concentration in CM‐ and ATPS‐isolated EVs after dextranase treatment (*n* = 7). (E) Recovery of EV subpopulations of ATPS‐isolated EVs with (right) or without (left) dextranase treatment (*n* = 7).

To improve the accuracy of immunolabelling and nFC analysis on EVs isolated by ATPS, dextranase was used to remove DEX in the EV‐containing DEX‐rich phase. nFC revealed CD9‐, CD63‐ and CD81‐positive EV subpopulations and biomarker/zsGreen double‐positive subpopulations with similar size distribution and signal intensity in dextranase‐treated samples, as in CM‐ and DEX‐rich phase without dextranase treatment (Figure 3A). EV concentration readings still demonstrated enrichment of lEVs (Figure 3B), sEVs (Figure 3C) and zsGreen/biomarker double‐positive subpopulations (Figure 3D) in dextranase‐treated samples, in comparison to EV subpopulation concentrations in CM. Calculation of recovery efficiency revealed that the presence of DEX in EV‐enriched DEX‐rich phase after ATPS leads to non‐specific immunolabelling and nFC reading, as the recovery efficiency exceeded 100% and reached 200% (Figure 3E). However, DEX removal with dextranase eliminates non‐specificity and reveals accurate immunolabelling and nFC analysis with near‐full recovery of biomarker‐positive subpopulations (Figure 3E).

### 3.4 Presence of Dextran Increases EV Internalization in Recipient Cells

**FIGURE 4:**
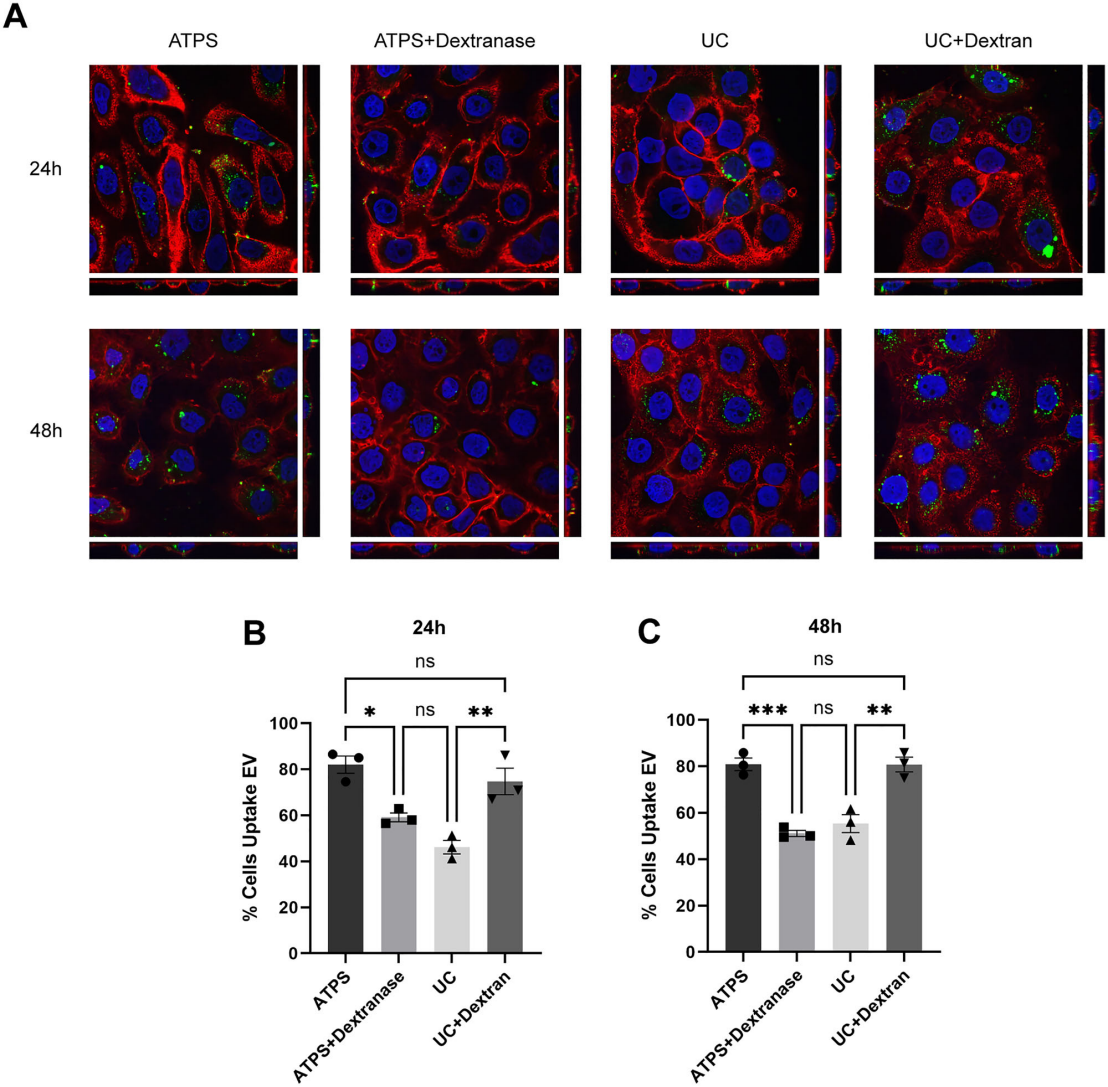
Presence of dextran improves EV internalization in recipient cells. (A) Confocal microscopy images and orthogonal views of BPH‐1 cells co‐cultured with ATPS‐isolated PC3‐zsGreen EVs, ATPS‐isolated PC3‐zsGreen EVs after dextranase treatment, UC‐isolated PC3‐zsGreen EVs, and UC‐isolated PC3‐zsGreen EVs mixed with dextran for 24 h (top panel) and 48 h (bottom panel). EVs were labelled with zsGreen (green). Recipient BPH‐1 cells were labelled with Hoechst 33,342 for nucleus (blue) and WGA Alexa Flour 647 for the plasma membrane (red). (B) Proportion of recipient cells showed EV internalization after 24‐h co‐culturing (*n* = 3). (C) Proportion of recipient cells showed EV internalization after 48 h co‐culturing (*n* = 3).

In addition to promising targets for biomarker and liquid biopsy development, EVs have also been found to participate in cell‐to‐cell communication for various physiological and pathological processes by horizontally transferring proteins and genetic materials between cells (Théry et al. 2018; Welsh et al. 2024; Das et al. 2024; van Niel et al. 2018). EV internalization assays are commonly used to study such EV functions by co‐culturing EVs with recipient cells *in vitro*. PC3‐zsGreen EVs isolated by ATPS and UC were co‐cultured with BPH‐1 cells to compare the recipient cell internalization efficacy. To study the impact of DEX on recipient cell internalization of EVs, in separate treatment groups, dextranase was used to remove DEX in ATPS‐isolated EVs, and DEX was mixed with UC‐isolated EVs to the same concentration as in DEX‐rich phase after ATPS. Confocal microscopy was used to visualize and quantify the internalization of PC3‐zsGreen EVs in BPH‐1 cells after co‐culturing for 24 h (Figure 4A, top panel) and 48 h (Figure 4B, bottom panel). Orthogonal views (*XZ* and *YZ*) revealed abundant cytosolic and minor membrane binding of EVs.

### 3.5 Next‐gen ATPS Offers Equivalent RNA Yield and Transcriptome Overlap Compared to Other Methods of RNA Isolation From EVs

**FIGURE 5:**
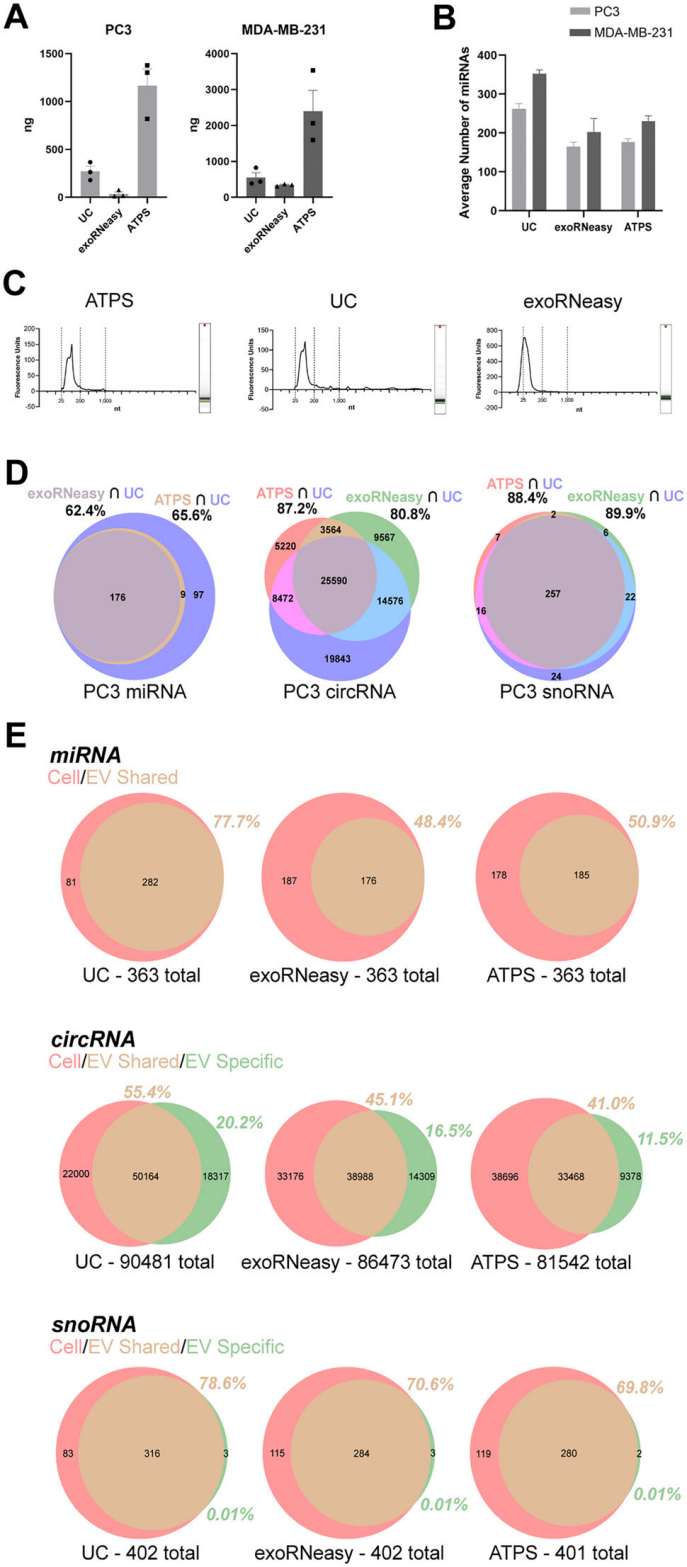
Transcriptomic profiles of CM EV RNA isolated by various methods. (A) The amount of total small RNA isolated from conditioned media of PC3 and MDA‐MB‐231 cells (*n* = 3). (B). Average number of miRNA species detected from next‐gen ATPS, UC and Qiagen exoRNeasy kit isolated EVs from conditioned media of PC3 (light grey bars, *n* = 2) and MDA‐MB‐231 cells (dark grey bars, *n* = 2). (C) Bioanalyzer profile of RNA extracted from EVs isolated by next‐gen ATPS (left), UC (middle) and Qiagen exoRNAeasy kit (right). EVs isolated from conditioned media of PC3 cells. (D) Venn diagrams describing the number of uniquely expressed and overlapped miRNA (left), circRNA (middle) and snoRNA (right) species across all three methods. (E) Venn diagrams describing the number of uniquely expressed and overlapped miRNA (top), circRNA (middle) and snoRNA (bottom) of all three methods compared to the small RNA species detected from the EV donor cells, PC3.

TRIzol‐chloroform separation followed by alcohol precipitation can be used in combination with UC or next‐gen ATPS to isolate RNA from EVs, but head‐to‐head comparisons to other kits that offer a complete solution were needed. Hence, the Qiagen exoRNeasy Midi Kit was also evaluated; this kit has an EV isolation column and a column for isolating the RNA from those EVs. The three methods were compared in isolating RNA from PC3 CM and MDA‐MB‐231 CM (Figure 5A,C). Yields were higher with the next‐gen ATPS‐TRIzol combination compared to the exoRNeasy and the UC‐TRizol combination in both CMs. A similar number of miRNAs was isolated by next‐gen ATPS and exoRNeasy in both CMs but the highest number was observed with UC‐isolated EVs (Figure 5B). Bioinformatics analysis was performed to determine the overlap of the miRNA, circular RNA (circRNA) and small nucleolar RNA (snoRNA) present across all three methods. In terms of miRNA and circRNA, next‐gen ATPS had a higher overlap with UC than exoRNeasy (Figure 5D, left and middle Venn diagram). Next‐gen ATPS and exoRNeasy were equivalent in terms of overlap with snoRNA (Figure 5D, right Venn diagram).

miRNA, circRNA and snoRNA isolated by the three different methods were compared to the EV donor cell source as shown in Figure 5E. The miRNA profile of parental cells exceeded the miRNA profile of EVs isolated by UC, exoRNeasy and next‐gen ATPS. UC EVs had the closest representation of parental cell miRNA; miRNA from exoRNeasy and next‐gen ATPS EVs had a lower representation of cell miRNA (Figure 5E, top panel). The circRNA profile of UC, exoRNeasy and next‐gen ATPS EVs was partially representative of the cellular circRNA profile but there was a subset of circRNAs specific to EVs (Figure 5E, middle panel). This was most pronounced in the UC Venn diagram (left) and least pronounced in the next‐gen ATPS Venn diagram (right). The snoRNA profiles were the smallest in number but all of the EV methods yielded a high representation of the parental cell snoRNA profile (Figure 5E, bottom panel).

### 3.6 Efficient Recovery of Patient Plasma EVs With Next‐gen ATPS

**FIGURE 6:**
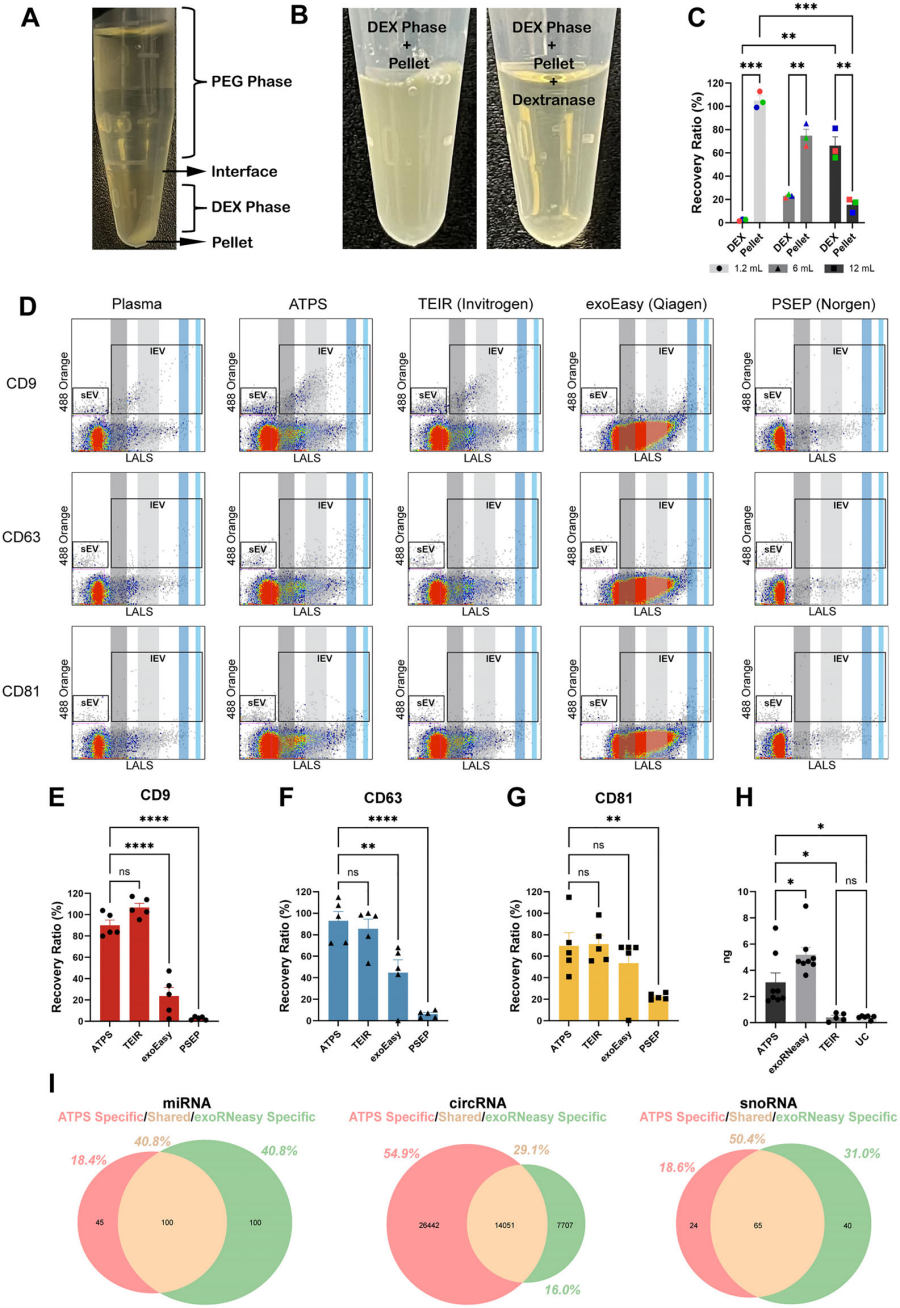
Comparison of EV isolation kit based on single event analysis with human prostate cancer plasma samples. (A) Appearance of a pellet in the DEX‐rich phase when using the 1.2‐mL version of ATPS. (B) Treatment of the DEX‐rich phase reduces pellet size. (C) Recovery ratios of EVs from both the DEX‐rich phase and the pellet when using 1.2‐, 6‐ and 12‐mL versions of the ATPS (*n* = 3). (D) Scatterplots showing single‐event analysis of isolated EVs from each isolation method using nFC. Isolation methods are shown across the left to right columns. Single event analysis for detection of CD9 (top row), CD63 (middle row) and CD81 (bottom row) +ve EVs. IEV, large‐size EVs (100–1400 nm). sEV, small EVs (<100 nm), dark grey area denotes LALS of 110‐nm calibration beads. Light grey area denotes LALS of 220‐nm calibration beads. Dark blue area denotes LALS of 800‐nm calibration beads. Light blue area denotes the LALS of 1400‐nm calibration beads. (E) Graph of EV concentration of CD9‐+ve EVs across all four EV isolation methods (*n* = 5). (F) Graph of EV concentration of CD63‐+ve EVs across all four EV isolation methods (*n* = 5). (G) Graph of EV concentration of CD81‐+ve EVs across all four EV isolation methods (*n* = 5). (H) Amount of small RNA from EVs isolated from the next‐gen ATPS method (*n* = 8), exoRNeasy kit (n = 8), total exosome isolation reagent (*n* = 5) and ultracentrifugation (*n* = 6). * denotes *p* < 0.01, Student's *t*‐test. (I) Transcriptomic profiles of miRNA (left panel), circular RNA (circRNA, middle panel) and small non‐coding RNA (snoRNA; right panel) present in EVs isolated via next‐gen ATPS versus exoRNeasy. The extent of transcriptomic overlap between the two methods is depicted in the beige intersection.

To confirm the applicability of using ATPS to isolate EVs for liquid biopsy development, human plasma samples from prostate cancer patients were subjected to ATPS. Although a sharp interface, upper PEG‐rich phase and lower DEX‐rich phase were observed, a pellet appeared when using certain volumes of the ATPS (1.2‐ and 6.0‐mL scale formats) with human samples, as a reflection of the complex composition of human plasma compared to cell culture conditioned media (Figure 6A). This pellet was isolated separately and dissolved in PBS after removing the DEX‐rich phase. Subsequently, the re‐suspended pellet and DEX‐rich phase were immunostained with PE‐conjugated antibodies against CD63 and analysed using nFC to ascertain the partitioning of plasma EVs in 1.2‐, 6.0‐ and 12.0‐mL scale formats of ATPS. Figure 6C illustrates the partitioning of nearly all EVs (∼100%) in the pellet when using the 1.2‐mL scale format of ATPS. EVs start to transit from the pellet to the DEX‐rich phase when increasing the total volume of ATPS to 6.0‐mL (∼23% in DEX‐rich phase and ∼74% in pellet) and 12.0‐mL scales (∼66% in DEX‐rich phase and ∼15% in pellet, Figure 6C). These findings suggest the importance of combining the pellet and DEX‐rich phase to accurately isolate total EVs when employing the 1.2‐mL scale format of ATPS on human plasma samples. Nonetheless, the pellet is reduced or eliminated after treatment with dextranase (Figure 6B).

Multiple commercialized EV isolation kits are available on the market, such as the Invitrogen TEIR, the Qiagen exoEasy Kit and the Norgen Plasma/Serum Exosome Purification Kit (PSEP). Human plasma samples were isolated with ATPS combined with dextranase treatment and commercially available EV isolation kits following the manufacturer's protocol, to compare their EV enrichment and recovery capabilities. To enumerate EVs in human prostate cancer plasma samples isolated by next‐gen ATPS and commercially viable kits, antibodies against EV biomarkers, CD9, CD63 and CD81 were used to label EV subpopulations for nFC analysis (Figure 6D). Next‐gen ATPS and TEIR revealed enriched CD9, CD63 and CD81 subpopulations, with size distribution and fluorescence intensity of EVs similar to findings with the original plasma samples. Plasma EVs isolated by exoEasy revealed biased EV subpopulations with large size and low fluorescence intensity (low antibody binding). This finding indicates that EVs obtained through exoEasy may not be ideal for nFC analysis. PSEP revealed minimal EV subpopulations, and if any were observed, were smaller in size, potentially due to the membrane filtration step in the manufacturer's protocol.

Figure 6E–G compares the recovery efficiency of CD9‐, CD63‐ and CD81‐positive EVs isolated by next‐gen ATPS against other commercially available kits. Next‐gen ATPS exhibited greater EV recovery compared to the exoEasy and PSEP. TEIR showed similar EV recovery as next‐gen ATPS. However, the recovery of CD9‐positive EVs isolated by the TEIR exceeded 100%, suggesting a minor level of non‐specific EV‐antibody binding due to the polymer precipitation method used by this kit.

Plasma EVs isolated by next‐gen ATPS, TEIR and UC were then submitted to TRIzol RNA isolation. To compare with column‐based methods, which are broadly used for EV RNA isolation, Qiagen exoRNeasy kit was also evaluated. While the exoRNeasy kit revealed the highest RNA yield, next‐gen ATPS in combination with TRIzol RNA isolation showed significantly higher RNA yield compared to TEIR and UC followed by TRIzol RNA isolation (Figure 6H). Human plasma EV RNA isolated by next‐gen ATPS and exoRNeasy kit was further submitted for small RNA sequencing, following the same pipeline for cell culture–derived EV small RNA sequencing, to further validate the application of next‐gen ATPS for biomarker and liquid biopsy discovery. Consistent with Figure 5, miRNA, circRNA and snoRNA were also detected in EVs isolated by next‐gen ATPS and exoRNeasy kit from human plasma samples (Figure 6I). The exoRNeasy kit revealed more miRNA (1.4 times) compared to the next‐gen ATPS, with a high overlapping of all miRNA species detected (Figure 6I, left). 1.9 times more circRNA species were detected from next‐gen ATPS isolated plasma EVs, highlighting the potential application of this method for circRNA‐based biomarker discovery (Figure 6I, middle). Both methods revealed a similar snoRNA profile (Figure 6I, right), and the highly overlapped small RNA profile for all three RNA types demonstrates that the next‐gen ATPS can reveal representative small RNA profiles from human samples.

Protein samples from human plasma starting material and EV preps post one round and two rounds of next‐gen ATPS were immunoblotted, revealing CD9 (EV marker) and Apolipoprotein A1 (ApoA1, high‐density lipoprotein marker), which is commonly reported in EV preps as protein contamination from human samples (Figure S4A). Degree of protein contamination, as represented by the expression level of ApoA1, was quantified by densitometry using the ImageJ software and normalized against CD9. While one‐step next‐gen ATPS showed reduction of ApoA1, two rounds of next‐gen ATPS further purify EVs from lipoprotein contaminations, reaching a reduction of 75%–90% (Figure S4B).

## 4 Discussion

We describe a next‐generation version of ATPS for the isolation of EVs from both *in vitro* cell culture media and human plasma samples. We compared this method against commercially available EV isolation kits and found that it was either comparable or superior in terms of EV yield and RNA yield. Isolated EVs by next‐gen ATPS were demonstrated to have increased uptake by recipient cells *in vitro* relative to those isolated by UC. Most importantly, the miRNA/circRNA/snoRNA transcriptome profile of EVs isolated by next‐gen ATPS shared high overlap with other methods such as the Qiagen exoRNAeasy kit. Lastly, the miRNA/circRNA/snoRNA transcriptome profiles for all EV‐isolation methods shared strong overlap with parental cell profiles, thus validating the small RNA extracted from EVs.

EV isolation methods have been extensively described by Kang et al. (2017), and ATPS is amongst the possible techniques available to users. There are three main advantages of current ATPS protocols for the isolation of EVs. The first is the use of minimal centrifugal force to pellet or isolate EVs, allowing users to use common benchtop centrifuges. Second, ATPS requires minimal use of plastics and uses affordable reagents to perform EV isolation. Third, ATPS requires significantly lower amounts of time relative to most kits and UC. Overall, these are important qualities that are required by users with high‐throughput EV isolation needs. The major disadvantage of current ATPS methods is that downstream use of these isolated EVs is hampered by the presence of DEX.

Here, our solution is to hydrolyse DEX by adding a small amount of dextranase. Within 10–15 mins, the majority of DEX is degraded and this enables standard RNA extraction techniques to be used such as TRIzol, a low‐cost reagent. The miRNA, circRNA and snoRNA profile of RNA extracted from EVs demonstrated that next‐gen ATPS leads to a similar transcriptomic EV profile as other methods. RNA from EVs represented the majority of the parental cell transcriptome, further validating the utility of the next‐gen ATPS method. It was not expected that the transcriptome of EVs isolated by next‐gen ATPS would eclipse or exceed that of EVs isolated by UC but at least it was equivalent or superior to the transcriptome of EVs isolated by other methods such as the Qiagen exoRNeasy kit. Moreover, the amount of RNA isolated is high, allowing users to proceed with confidence in downstream processes.

The next‐gen ATPS is also effective in isolating human plasma EVs and their RNA cargo and the results were equivalent or superior to commercially available EV isolation kits. While the RNA yields with the Qiagen exoEasy are high, these isolated EVs are not usable for other analyses such as flow cytometry. The TEIR (Invitrogen) is equivalent to the next‐gen ATPS method in terms of yield but EVs larger than 600 nm in diameter are less abundant compared to the next‐gen ATPS method. This suggests that the precipitation reaction that occurs when using the TEIR kit is inefficient in isolating the larger EVs. There is also a precipitation reaction that occurs with the next‐gen ATPS but in our protocol, the DEX phase and the pellet are both processed. Concerns about the pellet are minimized when the amount of ATPS polymers used exceed the initial volume of plasma being isolated. However, using an excessive amount of ATPS polymer will also decrease the concentration of isolated EVs. Therefore, a right balance of the ATPS polymer to the patient plasma sample must be found.

To our knowledge, the profiling of circRNAs within EVs and their profiling across different isolation kits and referencing this profile to the circRNA of the parental cell has not been performed before. CircRNAs are emerging as an important biomarker class and their presence within EVs isolated by next‐gen ATPS means that users can look forward to developing liquid biopsies with basic reagents such as TRIzol. snoRNAs were also observed in EVs isolated by the next‐gen ATPS method and with good overlap in snoRNAs isolated via UC and the exoRNAeasy kit.

There are several publications that describe the use of ATPS (Kim et al. 2015; Shin et al. 2018) for EV isolation and these are successful in isolating RNA from these EVs. Unfortunately, we were unable to reproduce these findings because of the DEX‐alcohol precipitates caused by the TRIzol reagent. It is not clear to us how the co‐precipitates were resolved by these investigators which forced us to use dextranase to hydrolyse the DEX. This improvement was essential in preventing precipitation and enabled us to achieve the expected RNA yields. This improvement was further corroborated by the transcriptomic analyses shown in Figure 5 whereby EVs isolated by the next‐gen ATPS method nearly eclipsed the transcriptome of UC‐isolated EVs. Another corroboration is that nearly half of the parental cell transcriptome was observed in next‐gen ATPS‐isolated EVs.

The impact of DEX was a concern for not only RNA isolation but also for immunolabeling with antibodies. It is unclear why DEX increases antibody binding to EVs but it is necessary to remove or degrade the DEX in order to perform immunolabeling experiments that use flow cytometry. This is not an issue for other techniques such as Western immunoblotting that separate the DEX from the proteins related to EVs. The use of dextranase was effective in reducing non‐specific binding caused by DEX and revealed that the next‐gen ATPS method provided the same type of EV subpopulation (CD9, CD61 and CD81) yields as expected when compared to the starting material. This is a key advantage because EVs isolated by the exoRNAeasy kit are exposed to various chemicals that alter the proteins in EVs, rendering them unusable for immunolabelling and hence flow cytometry analysis.

The presence of the 500‐kDa MW DEX led to an increased number of cells that endocytosed EVs from ∼60% to ∼80% of all cells. We are unaware of any reports demonstrating this increased uptake effect but this could be beneficial for users that are experiencing difficulties in attaining higher uptake rates across their cells of interest. If this is not required by the user, the use of dextranase to hydrolyse the DEX does restore uptake rates similar to EV isolated by conventional methods such as UC.

Plasma samples are a common biofluid that contain EVs, and various methods are known to have some inefficiencies when recovering biomarkers like small RNAs. The next‐gen ATPS when combined with TRIzol was adequate in isolating small RNA from prostate cancer patient plasma but when using the exoRNAeasy kit, higher yields were observed. This means that users who value both immunolabeling and RNA yield can consider the next‐gen ATPS method alongside the exoRNAeasy kit. However, if large amounts of plasma are available, then using the next‐gen ATPS kit and TRIzol may be just as effective and at the same time, achieved at a lower cost. One final advantage of the next‐gen ATPS method is that no additional equipment is needed for the isolation of EVs. In contrast, significant startup costs related to size exclusion‐based methods are needed for the isolation of EVs and columns for EV isolation may also augment the cost needed for EV isolation. Furthermore, the volume configurations for the next‐gen ATPS method can be scaled up in a straightforward and linear manner, which makes EV isolation for nanomedicine applications economical and fast. Overall, we present next‐gen ATPS as a solution that meets the needs of investigators who require a gentle and timely means of isolating EVs from cell culture media and plasma samples at a low cost. Next‐gen ATPS has wide‐ranging applications in other biofluids and samples, thus significantly de‐risking those investigations due to the low cost and ease of use. Finally, next‐gen ATPS is also a promising means of improving the robustness and reproducibility of biomarker‐based studies because it overcomes the variability of EV yield in UC. The small RNA transcriptomics data revealed the equivalence or superiority of the next‐gen ATPS method relative to other EV isolation techniques widely available. These are important findings that should factor into any practical implications when isolating EVs for downstream analyses.

## Author Contributions

Experimental design and investigation: Boyang Su, Morteza Jeyhani, Gobi Thillainadesan, Thamara Dayarathna, Scott S. H. Tsai and Hon S. Leong. Execution of experiments: Boyang Su, Morteza Jeyhani, Reese Wunsche, Minzhi Sheng, Hanyi Weng and Kristin Cimolai. Resources: Stanley K. Liu and Katarzyna J. Jerzak. Data analysis and interpretation: Boyang Su, Morteza Jeyhani, Gobi Thillainadesan, Thamara Dayarathna, Scott S. H. Tsai and Hon S. Leong. Preparation of manuscript and editing: Boyang Su, Morteza Jeyhani, Gobi Thillainadesan, Reese Wunsche, Thamara Dayarathna, Stanley K. Liu, Scott S. H. Tsai and Hon S. Leong.

## Conflicts of Interest

B.S., M.J., S.S.H.T. and H.S.L. are named co‐inventors on a provisional patent for the next‐gen ATPS method which has been assigned to their respective institutions (Toronto Metropolitan University and Sunnybrook Research Institute).

## Supporting information



Supporting Information

## Data Availability

The data that support the findings of this study are available from the corresponding author upon reasonable request. Small RNA sequencing data are openly available in NCBI GEO (Accession ID: GSE269052) at https://www.ncbi.nlm.nih.gov/geo/query/acc.cgi?acc=GSE269052 (Token: yhqzoeqwpxctrsf).
