# Supplementary material for: Next Generation Aqueous Two‐Phase System for Gentle, Effective, and Timely Extracellular Vesicle Isolation and Transcriptomic Analysis
Source: J Extracell Vesicles. 2025 Mar 19;14(3):e70058. doi: 10.1002/jev2.70058 (PMC11923243; doi:10.1002/jev2.70058)
Supplement: Supplementary file 1 — Supporting Information [file JEV2-14-e70058-s001.docx]

**Supplementary Table 1: Primers used in this study.**

| GAPDH_QPCR_F | ATGGGGAAGGTGAAGGTCG |
| --- | --- |
| GAPDH_QPCR_R | GGGGTCATTGATGGCAACAATA |

**Supplementary Table 2: Time for practice of EV isolation methods used in this study.**

| ATPS (for CM) | 20 minutes |
| --- | --- |
| ATPS (for plasma, including dextranase treatment) | 40 minutes |
| UC | 2.5 hours |
| Qiagen exoEasay Kit | 30 minutes |
| Norgen Plasma/Serum Exosome Purification Kit | 40 minutes |
| Invitrogen Total Exosome Isolation Reagent (for CM) | >12 hours |
| Invitrogen Total Exosome Isolation Reagent (for Plasma) | 30 minutes |

**
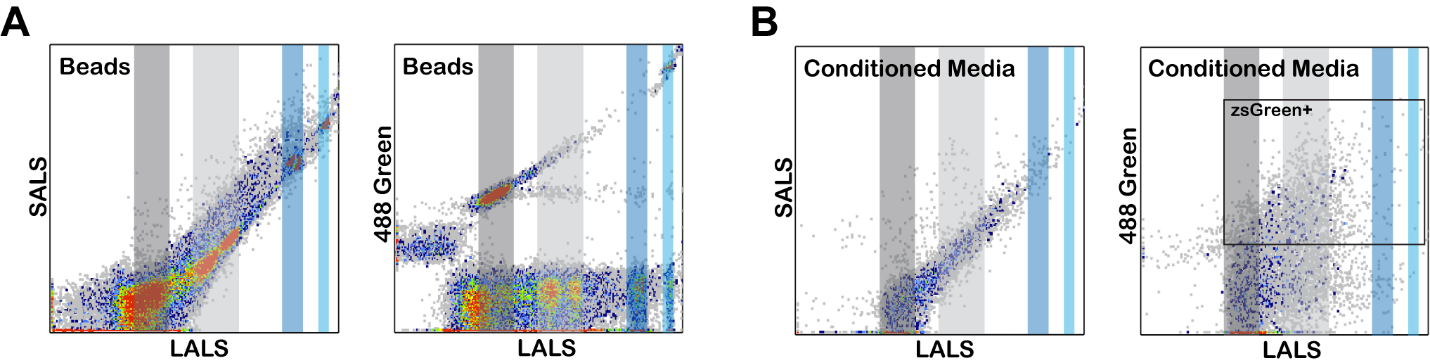
**

**Supplementary Figure 1. A60Micro-Plus nanoscale flow cytometer (nFC) calibration with beads and PC3-zsGreen conditioned media analysis.** A) nFC analysis of the ApogeeMix size calibration beads revealed size gates of 100-180 nm, 240-300 nm, 590-880 nm, and >1300 nm, indicated by shades from left to right. Results are plotted on LALS vs. SALS (left panel) and LALS vs. 488-Green (right panel) cytograms. B) nFC analysis of PC3-zsGreen conditioned media plotted on LALS vs. SALS (left panel) and LALS vs. 488-Green (right panel) cytograms. zsGreen positive EV subpopulation was gate


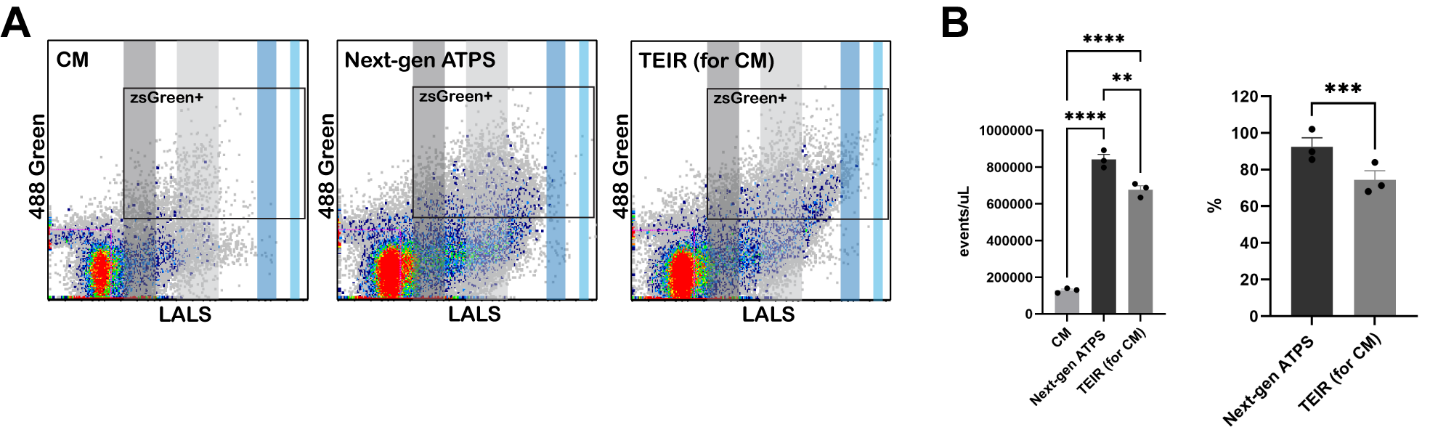


**Supplementary Figure 2. Comparison of ATPS and TEIR on CM EV Isolation.** A) nFC analysis of PC3-zsGreen CM (left), and EV preparation post ATPS (middle) and TEIR (right) isolation. Results are plotted on LALS vs. 488-Green cytograms. B) EV concentration (left, n=3) and recovery efficiency (right, n=3) of PC-zsGreen EVs isolated by ATPS and TEIR.

**
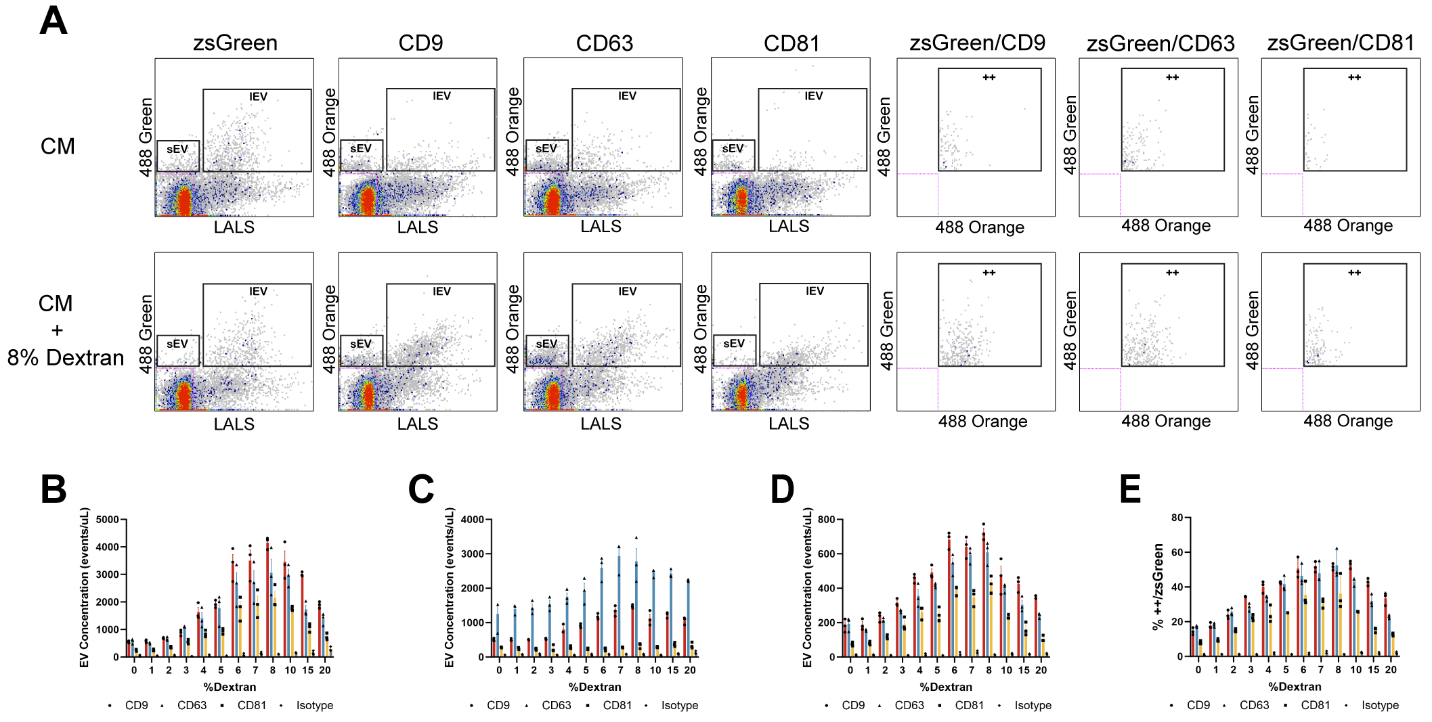
**

**Supplementary Figure 3. nFC analysis on the impact of dextran on EV-antibody labelling.** A). nFC anslysis of PC3-zsGreen conditioned media (top row) and PC3-zsGreen conditioned media mixed with 8% dextran (bottom row), labelled with PE-conjugated antibodies against CD9, CD63 and CD81. Cytograms show zsGreen1 positive EV subpopulations (1^st^ panel), biomarker positive EV subpopulations (2^nd^ to 4^th^ panel), and zsGreen1/biomarker double positive EV subpopulations (5^th^ to 7^th^ panel). B). CD9, CD63, CD81 positive large EV subpopulation concentration of PC3-zsGreen conditioned media mixed with 0%, 1%, 2%, 3%,4%, 5%, 6%, 7%, 8%, 10%, 15%, 20% dextran (n=3). C). CD9, CD63, CD81 positive small EV subpopulation concentration of PC3-zsGreen conditioned media mixed with the same dextran gradient (n=3). D). zsGreen/biomarker double positive EV subpopulation concentration of PC3-zsGreen conditioned media mixed with the same dextran gradient (n=3). E). Proportion of zsGreen/biomarker double positive EV subpopulations in total zsGreen positive subpopulation of PC3-zsGreen conditioned media mixed with the same dextran gradient (n=3).


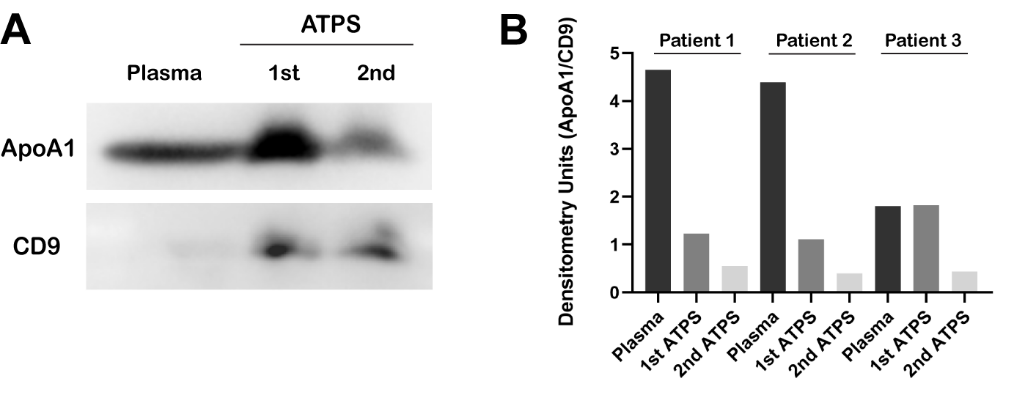


**Supplementary Figure 4. Degree of protein contamination in EV prep post next-gen ATPS.** A). Immunoblot of Apolipoprotein A1 (ApoA1) and CD9 in prostate cancer human plasma samples, EV isolated by one-step next-gen ATPS, and two-step next-gen ATPS. B). Analysis of ApoA1 expression normalized against CD9 from three patients.
